# Adenovirus Type 7 causing severe lower respiratory tract infection in immunocompetent adults: a comparison of two contrasting cases from an intensive care unit in North West England^☆^^☆☆^

**DOI:** 10.1016/j.clinpr.2019.100007

**Published:** 2019-10

**Authors:** Tom Wingfield, Luke Dearden, Pete Calvert, Orod Osanlou, Brian Johnston, Anu Chawla, Ian Hart, Catherine Thompson, Lance Turtle, Richard Wenstone

**Affiliations:** a LIV-TB Collaboration and Departments of Clinical Sciences and International Public Health, Liverpool School of Tropical Medicine, Liverpool, UK; b Department of Clinical Infection, Microbiology and Immunology, Institute of Infection and Global Health, University of Liverpool, UK; c Tropical and Infectious Diseases Unit, Royal Liverpool University and Broadgreen Hospitals NHS Trust, Liverpool, UK; d Social Medicine, Infectious Diseases and Migration Group, Department of Public Health Sciences, Karolinska Institutet, Stockholm, Sweden; e Intensive Care Unit, Royal Liverpool University and Broadgreen Hospitals NHS Trust, Liverpool, UK; f Department of Clinical Pharmacology, Royal Liverpool University and Broadgreen Hospitals NHS Trust, Liverpool, UK; g Liverpool Specialist Virology Centre, Royal Liverpool University and Broadgreen Hospitals NHS Trust, Liverpool, UK; h Respiratory Virus Unit, Virus Reference Department, National Infection Service, Public Health England, 61 Colindale Avenue, London NW9 5EQ, UK

**Keywords:** Severe lower respiratory tract infection, Pneumonia, Pneumonitis, Acute respiratory distress, Adenovirus, Critical care, Intensive care

## Abstract

**Objectives:**

Severe lower respiratory tract infection caused by adenovirus is well described in immunocompromised hosts and can cause significant morbidity and mortality. We compare and contrast the clinical presentation, radiological, and virological features of two rare cases in immunocompetent adults admitted to an intensive care unit in a large, teaching hospital in North West England. We then provide a concise, comprehensive literature review.

**Methods:**

The first case was a 35-year old female asthmatic who presented with respiratory distress and pneumonitis during peak influenza season, and recovered after a prolonged hospital stay. The second case was a 73-year old male who presented with diarrhoea, vomiting, and general malaise outside of influenza season, developed respiratory compromise, and died. Adenovirus type 7 was identified in bronchoalveolar lavages and plasma samples of both patients, each of whom received cidofovir. No other infectious aetiology was identified.

**Results:**

Clinical and radiological features of severe lower respiratory tract adenoviral infection are similar to other infectious causes of pneumonia and ARDS, including severe influenza. This can create diagnostic uncertainty, especially during influenza season. Positive adenovirus polymerase chain reaction results can support a diagnosis of severe lower respiratory tract adenovirus infection in patients with a clinically compatible syndrome and no other identified aetiology, with higher viral loads being associated with worse prognosis. Although treatment is predominantly supportive, early use of cidofovir may improve outcomes.

**Conclusions:**

These rare cases highlight that severe lower respiratory tract adenoviral infection should be considered in the differential diagnoses of immunocompetent patients presenting with pneumonia and ARDS.

## Highlights

• Severe lower respiratory tract adenovirus infection requiring critical care admission is rare in immunocompetent adults.
• Evidence concerning diagnosis and management of immunocompetent adults with adenovirus severe lower respiratory tract infection (sLRTI) is limited.
• We present rare cases of severe lower respiratory tract adenovirus infection in immunocompetent adults, on a critical care unit.
• Immunocompetent patients with adenovirus sLRTI may present with pneumonitis and acute respiratory distress syndrome (ARDS)
• Clinical features of sLRTI are difficult to distinguish clinically and radiologically from other causes of pneumonitis, including severe influenza.

## Background

Severe lower respiratory tract infection (sLRTI) due to adenovirus is well described in immunocompromised hosts. Adenovirus sLRTI is rare in immunocompetent hosts, especially adults, and evidence regarding symptoms, diagnosis and management in such instances remains limited. Here, we describe two cases of severe adenovirus in immunocompetent adults admitted to an intensive care unit in a large, teaching hospital in North West England.

## Case 1

In January 2018, a 35-year-old homeless female commercial sex worker presented with a 4-day history of pharyngitis, myalgia, cough productive of green sputum, and dyspnoea. She was asthmatic but took no regular medications and denied related hospital admissions. Other medical history included obesity, schizophrenia, personality disorder, depression and self-harm. She was a smoker, did not take alcohol or drugs and had no fixed abode.

Initial evaluation revealed widespread bilateral rhonchi and severe respiratory distress. A diagnosis of a life-threatening asthma exacerbation precipitated by LRTI was made. Antimicrobial treatment commenced included oseltamivir (due to presentation during peak of influenza season and risk factors for viral pneumonitis including chronic lung disease and obesity), benzylpenicillin and clarithromycin. Respiratory distress was treated with oxygen therapy, nebulised bronchodilators, intravenous steroids and magnesium. Investigations showed lymphopenia with raised inflammatory markers but normal liver and kidney function. Arterial blood gas showed type 1 respiratory failure. Chest radiograph showed bilateral patchy consolidation. Blood-borne virus (BBV) screen, including HIV, was negative.

**Fig. 1:**
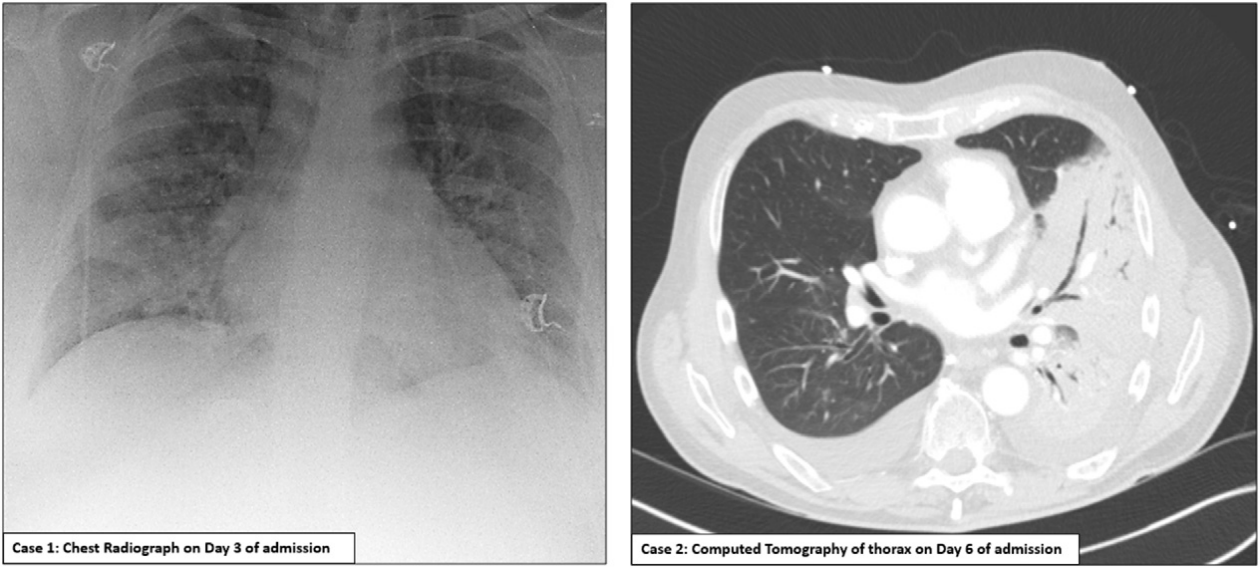
Radiological findings in Case 1 versus Case 2.

She deteriorated over 48 h with increasing drowsiness and respiratory distress. Chest radiograph showed increasing bilateral consolidation (Fig. 1) and antibiotics were broadened to piperacillin/tazobactam. On day 3, she was transferred to the High Dependency Unit (HDU, Level-2 Care) for respiratory support. Aminophylline and salbutamol infusions and CPAP were instigated but she deteriorated and was transferred to the intensive care unit (ICU, Level-3 Care) for intubation and ventilation.

Following intubation, bronchoscopy with diagnostic bronchoalveolar lavage (BAL) was performed. Despite paralysis and airway pressure release ventilation for respiratory recruitment, the patient continued to desaturate and developed type 2 respiratory failure. In an attempt to improve ventilation, inhaled nitric oxide was commenced and the patient turned into the prone position. On day 8, BAL returned a strongly positive adenovirus by polymerase chain reaction (PCR) test (Table 1) and twice weekly cidofovir (with a loading dose one week after the initial dose) was commenced. Renal replacement therapy was started on day 12 for renal failure and fluid removal. By day 20, the patient had improved enough to allow tracheostomy to facilitate respiratory weaning and cessation of sedation. On day 52, the patient no longer required ventilation and, by day 65, she was stepped down to ward-level care.

**Table 1:** Clinical features, investigations, treatment and outcome of two cases of adenovirus sLRTI.

Case	Comorbidities	Presentation	Initial blood results	Radiological findings	Virology results	Adenovirus treatment given & AEs	Outcome
Case 1: 35 year old female	Asthmatic, obese, mental illness	4 day history of URTI and LRTI symptoms Presented in peak influenza season	Lymphopenia, mildly raised CRP No initial renal dysfunction^a^	CXR: bilateral consolidation, worsening during admission (Fig. 1)	Throat swab: adenovirus PCR positive BAL: adenovirus PCR positive at 5.8 × 10^8^ gEq/ml EDTA plasma: adenovirus PCR positive at 9.5 × 10^6^ gEq/ml^b^ Adenovirus species B type 7 by genomic sequencing	Cidofovir – renal impairment requiring RRT Consideration for ECMO but not initiated due to improvement	Decannulated on day 52 and stepped down to ward level care on day 65
Case 2: 73 year old male	Hypertension, nephrolithiasis	3 day history of generalised and gastrointestinal symptoms Presented outside of influenza season	Lymphopenia, mildly raised CRP No initial renal dysfunction^a^	CT chest: left-sided pneumonia and bilateral pleural effusions (Fig. 1)	Throat swab: adenovirus PCR positive BAL: adenovirus PCR positive at 7.5 × 10^9^ gEq/ml EDTA plasma: adenovirus PCR positive at 2.0 × 10^6^ gEq/ml^b^ Adenovirus species B type 7 by genomic sequencing	Cidofovir – renal impairment requiring RRT IVIG also given Consideration for ECMO but not initiated due to futility.	Died on day 12 due to multi-organ failure

**Abbreviations**: BBV = blood borne viruses; LRTI = lower respiratory tract infection; HIV, human immunodeficiency virus; CRP = C-reactive protein; CXR = chest radiograph; CT = computed tomography; BAL = bronchoalveolar lavage; RRT = renal replacement therapy; ECMO = extra-corporeal membrane oxygenation; gEq/ml = genome equivalents/millilitre, IVIG = intravenous immunoglobulin; HDU = high dependency unit (Level 2 Care), URTI = upper respiratory tract infection.

a Other initial blood tests including haemoglobin, platelets, clotting profile, liver function tests, and bone profile were normal.

b Full infection screen included blood cultures, legionella/pneumococcal urinary antigens, urine bacterial culture, blood-borne virus (e.g. HIV, Hepatitis B, and Hepatitis C), serum EDTA plasma: EBV PCR (positive in Case 1 at 728 copies gEq/ml and positive Case 2 at 908 copies gEq/ml, log 3), CMV PCR negative in Cases 1 and 2, upper respiratory tract bacterial culture and extended viral PCR panel, sputum microscopy and bacterial culture and extended viral PCR panel (including from BAL samples), faecal bacterial culture and *Clostridium difficile* screening, and resistant organism swabs including VRE and CPE (Case 2 was VRE positive).

## Case 2

In April 2018, a 73-year-old male presented with a 3-day history of diarrhoea, vomiting, malaise, myalgia and lethargy without respiratory symptoms. He was a non-smoker who had amlodipine-controlled hypertension and nephrolithiasis but was normally well and regularly hill-walked, including an extended 3-day hike just prior to admission.

Examination was unremarkable apart from hypotension (92/70 mmHg) and pyrexia (38.2 °C). He was lymphopenic but had normal liver and renal function. A chest radiograph demonstrated left middle-zone consolidation. He was diagnosed with community-acquired pneumonia and benzylpenicillin and clarithromycin were started.

By day 2, he had deteriorated with type 1 respiratory failure. Benzylpenicillin was switched to piperacillin/tazobactam and he was transferred to HDU. By day 4, there was no improvement and antibiotics were empirically changed to meropenem, doxycycline and linezolid. By day 6, he required transfer to ICU for intubation and ventilation. A chest CT demonstrated left-sided consolidation with bilateral pleural effusions (Fig. 1) and he underwent bronchoscopy with BAL. On day 8, throat swab, BAL and EDTA plasma adenovirus PCRs were strong positives (Table 1) and a single dose of cidofovir was given. No other bacterial or viral pathogens were isolated and BBV screen, including HIV, was negative.

By day 10, his respiratory compromise worsened and he was turned into the prone position. He became increasingly haemodynamically unstable and developed multi-organ failure including deteriorating renal function requiring continuous haemofiltration. A dose of normal human immunoglobulin was given. On day 12, life sustaining treatment was withdrawn.

## Discussion

Human adenoviruses are non-enveloped DNA viruses^1^ of the *Adenoviridae* family grouped into 7 species (A to G) consisting of over 85 known genotypes. They are associated with infections of the conjunctiva, respiratory and gastrointestinal epithelial tissues, and less commonly with haemorrhagic cystitis, haemorrhagic colitis, hepatitis, pancreatitis, nephritis, or encephalitis.^2^ sLRTI and disseminated disease are well recognised in immunocompromised patients^3^ but uncommon in immunocompetent hosts, especially immunocompetent adults.^2^ A multicentre study of 800 immunocompetent adult and child patients with viral LRTIs identified adenovirus as the cause in only 2%.^4^

The clinical and radiological features of adenovirus sLRTI are similar to sLRTI of other infectious aetiology, which can lead to diagnostic uncertainty. In reported outbreaks in immunocompetent people,2., 5., 6., 7. similar to recent influenza epidemics, fever, cough, and myalgia were the most common symptoms, followed by upper respiratory tract (rhinorrhoea and nasal congestion)^6^ and gastrointestinal (diarrhoea and nausea) symptoms. Predominant findings on clinical examination are also not discriminatory and include fever, chest signs (e.g. crepitations), and hypoxia.^7^ Rarely, patients can develop acute respiratory distress syndrome (ARDS).^8^ With regard to diagnosis, standard blood tests are often unremarkable but liver function tests may be abnormal and total leucocyte count can be decreased initially9., 10., 11.; one review of 21 immunocompetent patients with adenovirus pneumonia found lymphopenia in 11 (52%) and raised transaminases in 6 (29%).^7^ Similarly, reviews including the radiological features of adenovirus-related lung infection and ARDS have found that the majority of patients had multifocal, diffuse, bilateral parenchymal infiltrates including ground-glass on CT while approximately one quarter had lobar consolidation.7., 11. With regard to our patients, Case 1 presented during England's influenza season with respiratory symptoms and signs of pneumonitis followed by bilateral consolidation; Case 2 presented with generalised and gastrointestinal symptoms following influenza season and was found to have unilateral pneumonia and pleural effusions (Fig. 1). Ultimately, from reflection on these cases and review of the literature, there appear to be no clinical features specific to adenovirus sLRTI. This means that, for admitting clinicians, a careful review of the patient's history and identification of salient epidemiological risk factors (e.g., housing, occupation, known local outbreaks) remain of paramount importance.

Laboratory diagnosis of adenoviral infection can be challenging. The clinical utility of viral cultures for identification of adenovirus is limited by time to positivity (≤ 7 days) and antigen testing has low sensitivity and specificity.^2^ Therefore, adenovirus identification is best achieved using PCR. However, a positive PCR result does not equate to aetiology and must be evaluated in the clinical context.^2^ Both of our patients had high adenoviral loads and viraemia, which, in the context of confirmed adenoviral sLRTI in immunocompetent children and adults, are associated with worse prognosis and higher mortality.^12^ Our patients' viraemia could also suggest disseminated adenoviral disease but no relevant samples (e.g. tissue biopsy) were taken to confirm this.

**Fig. 2:**
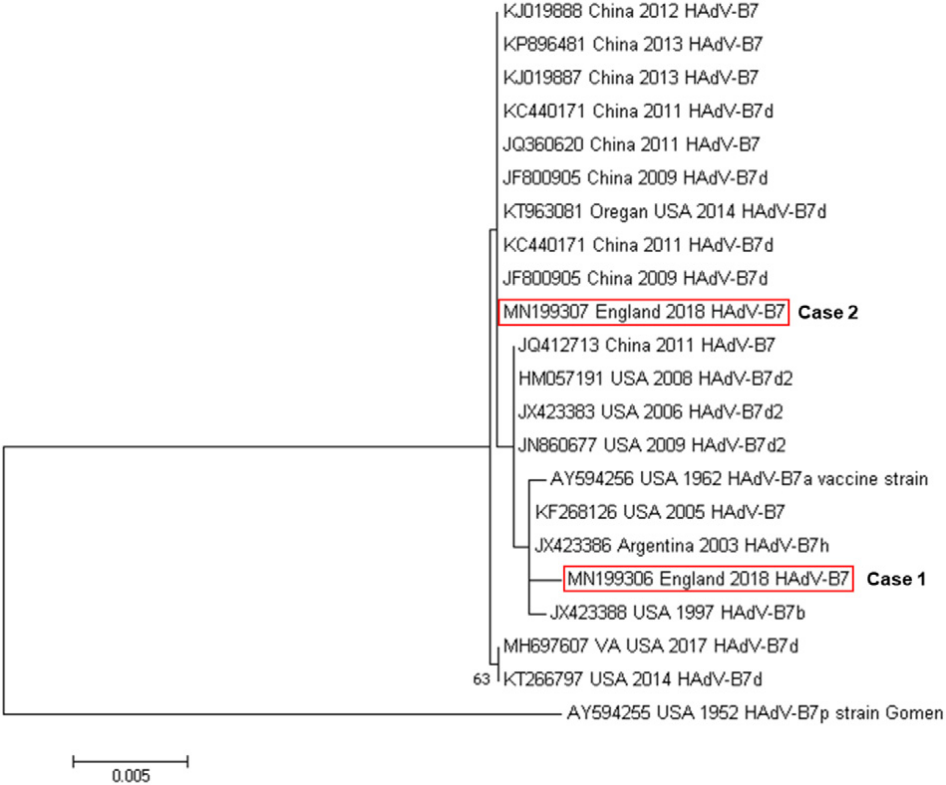
Evolutionary relationship of Human Adenovirus type B7 (HAdV-B7) partial hexon gene sequences including sequences obtained from two cases of adenovirus sLRTI. Legend: The maximum likelihood phylogenetic tree was constructed using the Tamura 3-parameter model in MEGA7 and is mid-point rooted.16., 17. Bootstrap values (> 50%) are shown for key nodes. The tree is drawn to scale, with branch lengths measured in the number of substitutions per site. Scale bar indicates estimated number of nucleotide substitutions per site. Sequences are identified by GenBank accession number, geographic location, year of sample collection, and virus genome type identified. Sequences obtained from cases described in this study are highlighted: Human Adenovirus (HAdV) strains Human\England\05.1\2018\B7 (MN199306) and Human\England\21.1\2018\B7 (MN199307).

To identify adenovirus type in our cases, two PCR assays targeting different regions of the adenovirus genome were used to amplify PCR products from BAL samples, two regions of the adenovirus hexon gene were sequenced using Sanger dideoxy sequencing methods, and these were then compared using BLAST for similarity to known adenovirus sequences in national databases.13., 14., 15. Partial hexon gene sequences (1436 bp) obtained from one PCR assay covering the hypervariable region were compared with HAdV-B7 reference strains available in GenBank (this study GenBank accession numbers MN199306, MN199307). Sequences were aligned using ClustalW embedded in BioEdit (V 7.0.5.3.), and a maximum likelihood phylogenetic tree was constructed using the Tamura 3-parameter model in MEGA7 (Fig. 2).16., 17. DNA extraction from all samples was done using the QIAsymphony DSP DNA Kit on the QIAsymphony instrument (QIAGEN Instruments AG, Hombrechtikon, Switzerland), the adenovirus quantitative PCR method used was the one described by Heim et al.,^13^ and run on the LightCycler 480 Instrument (Roche, Basel, Switzerland).

Both of our patients had adenovirus type 7, which was not associated with any known, local outbreaks, but has been implicated in outbreaks in the literature: adenovirus types 3 and 7 were isolated from 541/800 previously-healthy individuals with acute respiratory illness in a military barracks^6^; and of 198 patients with proven adenovirus LRTI in Oregon,^5^ one third had adenovirus type 7, which was also associated with higher morbidity than non-type 7. Partial hexon gene sequences (hypervariable region) from the two cases described here showed 99.7% similarity to each other. Sequence from Case 2 clustered together with recent HAdV-B7d sequences from China and the Oregon LRTI patients. Sequence from Case 1 did not form part of this cluster (Fig. 2). Although sequencing of the hexon hypervariable region is strongly correlated with virus serotype, as these were only partial hexon gene sequences, whole genome sequencing would be required to provide full genomic detail, to determine whether recombination had occurred and definitively define the genotype. It has been postulated that military recruits are at increased risk of adenovirus infection due to crowding and also the physical and psychological stress associated with their training.^6^ In addition, it has been suggested that obesity may be associated with adenovirus disease in immunocompetent individuals although the exact mechanism behind this remains unclear.^18^ Therefore, adenovirus type 7 may have been the cause of the severity of illness seen in our patients but equally it could have related to obesity, chronic lung disease, smoking, and social risk factors including unstable housing as external stressors (Case 1) and advanced age and comorbidities coupled with extensive physical exertion shortly prior to admission during an extended hike (Case 2).

Randomised-controlled trial evidence for antiviral treatments in adenovirus infection is lacking^19^ with the mainstay of care being supportive, including ECMO and renal replacement where necessary.^20^ Cidofovir has been shown to reduce adenoviral loads in a case series of 7 patients (all of whom survived) but its use, as in our patients, is limited by nephrotoxicity.^21^ Brincidofovir, a lipid ester of cidofovir, is an emerging alternative adenovirus therapy that does not cause nephrotoxicity and is currently undergoing randomised controlled evaluation.^22^ There is no evidence for IVIG in adenovirus infections but it was given to Case 2 due to biologically-plausible benefit of providing antibodies directed against the virus.

## Conclusions

Severe lower respiratory tract infection with adenovirus, especially type 7, can cause significant morbidity and mortality. Clinical and radiological features are similar to other infective causes of LRTI, pneumonia and ARDS (including severe influenza) but, as in Case 2 and similar to recent influenza epidemics, presenting symptoms can be non-specific. Positive adenovirus PCR can support a diagnosis in patients with a clinically compatible syndrome and no other identified aetiology, and higher viral loads are associated with worse prognosis. Although treatment is predominantly supportive, early use of cidofovir may improve outcomes. These two rare cases highlight that adenovirus sLRTI should be considered in the differential diagnoses of immunocompetent adults presenting with sLRTI, pneumonia, and/or ARDS.
